# Holding onto power: why confidence intervals are not (usually) the best basis for sample size calculations

**DOI:** 10.1186/1745-6215-12-S1-A101

**Published:** 2011-12-13

**Authors:** Chris Metcalfe

**Affiliations:** 1 School for Social and Community Medicine, University of Bristol, Canynge Hall, 39 Whatley Road, Bristol, BS8 2PS, UK

## Objectives

It has recently been suggested in a high profile paper that statistical power is no longer a useful basis for sample size calculations (Bland, BMJ 2009). It is proposed instead to calculate the sample size to achieve a narrow confidence interval width for the treatment effect estimate. My objective is to critically appraise this proposal.

## Methods

I compare the proposed approach to sample size calculations with the traditional statistical power based approach, and to the sample size calculations employed for equivalence studies which are also based on confidence interval width.

## Results

With a little simplification, the sample size calculations for the traditional power-based approach, for equivalence studies, and following the new proposal can be shown to be much the same. The single fundamental difference is that the new proposal does not include a multiplier to increase the statistical power beyond 50% (i.e. only a 50:50 chance of detecting a true treatment effect of clinically important magnitude). The attempt to avoid having to define a minimum clinically important difference on a predefined primary outcome is wholly unsuccessful. The calculation of confidence interval width must be based on a particular outcome measure, still requires the size of an unimportant difference to be defined if the confidence interval is to exclude it, and additionally requires a likely true effect of treatment to be defined about which the confidence interval will be centred.

## Conclusions

The proposal to base all sample size calculations on confidence interval width does not avoid the need to pre-define the minimum clinically important difference on particular important outcome measures, and in fact additionally requires that the likely effect of the intervention is specified. Most importantly, the approach does not replace statistical power. Statistical power is simply an inflation of the sample size to allow a good chance that a true treatment effect of clinically important magnitude will be detected, even if by chance it is underestimated in the trial data (as it will be, even if only slightly, with 50% probability). I conclude that statistical power is not the source of dissatisfaction with sample size calculations, and there is no real need to replace it as the basis for sample size calculations.

